# Supplementary material for: Horizontal face information is the main gateway to the shape and surface cues to familiar face identity
Source: PLoS One. 2024 Oct 7;19(10):e0311225. doi: 10.1371/journal.pone.0311225 (PMC11458052; doi:10.1371/journal.pone.0311225)
Supplement: S1 File — All figures and analysis cited in the manuscript are in this file. (DOCX) [file pone.0311225.s001.docx]

# Supporting Information

S.1 - Image selection pre-test

For the image selection pilot, 237 subjects, mean age = 25.3 ± 4.3 were recruited via social media and gave their informed written consent to do the task (no compensation was given but participants could enroll in a lottery to win 10 euros every 10 participants). We had 10 pictures for each of the 21 male actors we selected (210 images in total). Participants viewed a total of 70 randomly selected images (between three and four per actor). Each image was presented on a grey background for 500ms. Participants then had to click on the name of the actor among five names or click on ‘I don’t know’. After analysis, three actors were taken out of the set as more than half of the answers for their pictures were ‘I don’t know’. For the remaining 18 actors, the four best recognized images were selected. Three to be used during the trials of the experiment. The last image was kept to remind the different identities to the participants during the breaks, without exposing them to the experimental stimuli. To ensure the image was the most representative of all 4 images we calculated the correlation between each of the 4 images and a mean image of the actor made from all of his pictures on Matlab R2014a, and took the one with the highest score. The 18 selected actors are Ben Affleck, Ben Stiller, Brad Pitt, Daniel Craig, Daniel Radcliffe, Elijah Wood, George Clooney, Hugh Jackman, Justin Timberlake, Keanu Reeves, Leonardo DiCaprio, Matt Damon, Orlando Bloom, Patrick Dempsey, Robert Downey Jr., Robert Pattinson, Ryan Gosling, Ryan Reynolds

**
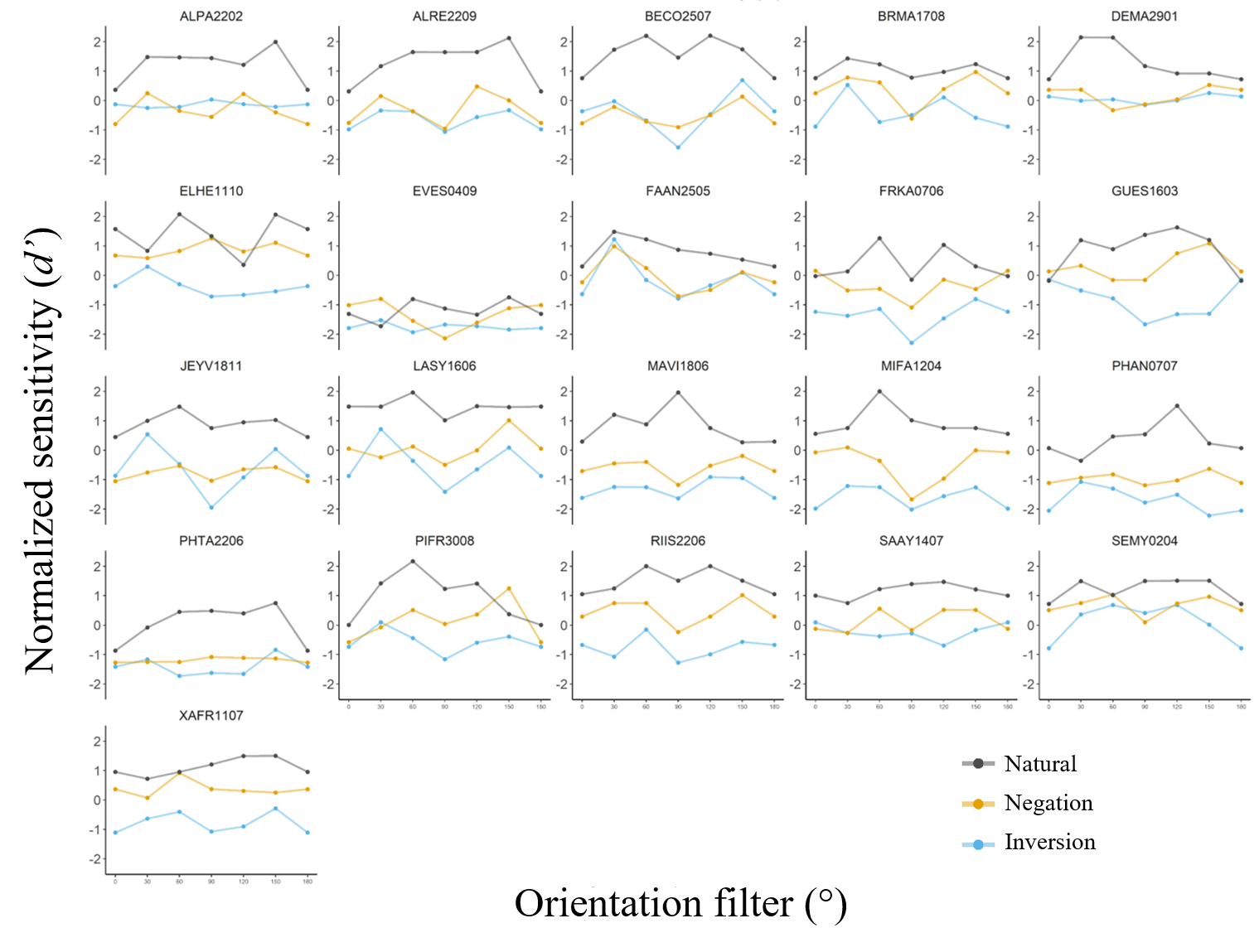
**S.2 - Individual orientation tuning for each Stimulus type

**
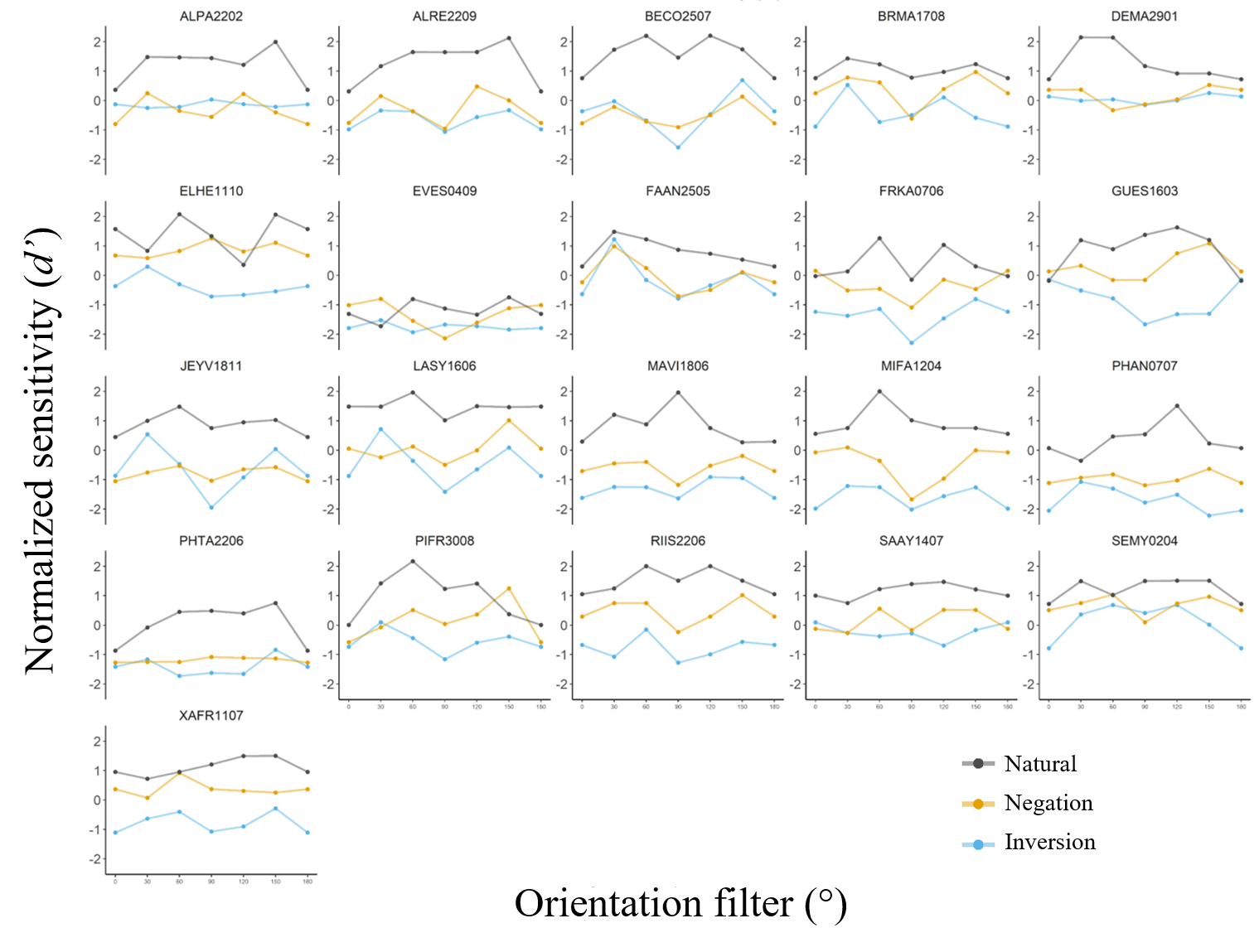
**

**Figure S.1.** Recognition performance (*normd’*) as a function of the Orientation filter for each Stimulus type (grey: Natural faces; yellow: Negated faces; blue: Inverted faces) and each participant.

S.3 - Partialling out the contribution of the performance in the Natural face condition to correlations between inversion and negation effects

The correlations between the Gaussian parameter estimates of the inversion and the negation effects were high and significant. Because we obtained the effects by subtracting the sensitivity under Inverted or Negated conditions from the sensitivity for Natural images, at each orientation filter, the Natural condition is common to both effect and could explain the correlation between inversion and negation (DeGutis et al., 2013). To determine whether it is the case or not, we ran partial Pearson correlations between the inversion and negation effects parameter estimates while using the parameter estimates of the model of the Natural condition (Supplementary 4) as controlling variables. If the shared variance of the orientation tuning profile of the inversion and negation effects reflect the use of sensitivity in the Natural condition as a common reference, the coefficient of the partial correlations should drop compared to the correlations reported in the main text.

Results of these partial Pearson correlations showed similar correlations (in terms of value, significance and confidence interval, see Table S.1) between the effects of inversion and negation when controlling for the Natural condition or not. This was the case for the Standard deviation estimate (r = .79, p <.0001), the Peak amplitude estimate (r = .99, p <.0001), the Peak location estimate (r = .99, p <.0001) and the Base amplitude estimate (r = .6, p<.01). These results show that the use of the Natural condition to compute the effects of inversion and negation does not explain the high correlations we measured between the effects of inversion and negation on those parameters.

**Table S.1**: Pearson correlations values, p-values and 95% confidence interval between the parameter estimates of the inversion and negation effect as reported in the manuscript (left) and partial correlation using the parameter estimates of the Natural condition.

| **ParameterEstimate** | **Cor** | **Pvalue** | **CI_low** | **CI_High** | **Par.Cor.** | **Pvalue** | **CI_low** | **CI_High** |
| --- | --- | --- | --- | --- | --- | --- | --- | --- |
| Base Amplitude | .7192 | p < .001 | [ .4170 | .8782 ] | .5996 | p = .005 | [ .2265 | .8192 ] |
| Peak Amplitude | .9863 | p < .001 | [ .9657 | .9945 ] | .9783 | p < .001 | [ .9462 | .9913 ] |
| Standard Deviation | .7815 | p < .001 | [ .5279 | .9072 ] | .7891 | p < .001 | [ .5320 | .9106 ] |
| Peak Location | .9967 | p < .001 | [ .9917 | .9987 ] | .9871 | p < .001 | [ .9677 | .9948 ] |

S.4 - Orientation tuning profile for the recognition of faces in the Natural condition

To compare the orientation tuning of face recognition obtained in the Natural condition to the results obtained in previous studies, we modelled normalized sensitivity (*normd’*) obtained in this experimental condition with a Gaussian model. We used the exact same formula and settings as the one used to model the inversion and negation effects (see Methods – Data Analysis). Namely, we ran the model with default *brms* priors with four Markov chain Monte Carlo (MCMC) with 6,000 iterations each (3,000 warm up, resulting in 12,000 iterations after warmup), and evaluated the fit with diagnostic values such as the number of divergent chains post warmup, the Rhat statistics and the effective sample size the convergence of the chains. Model and data are shown in Figure S.2, the parameters estimated by the model are reported in Table S.2.


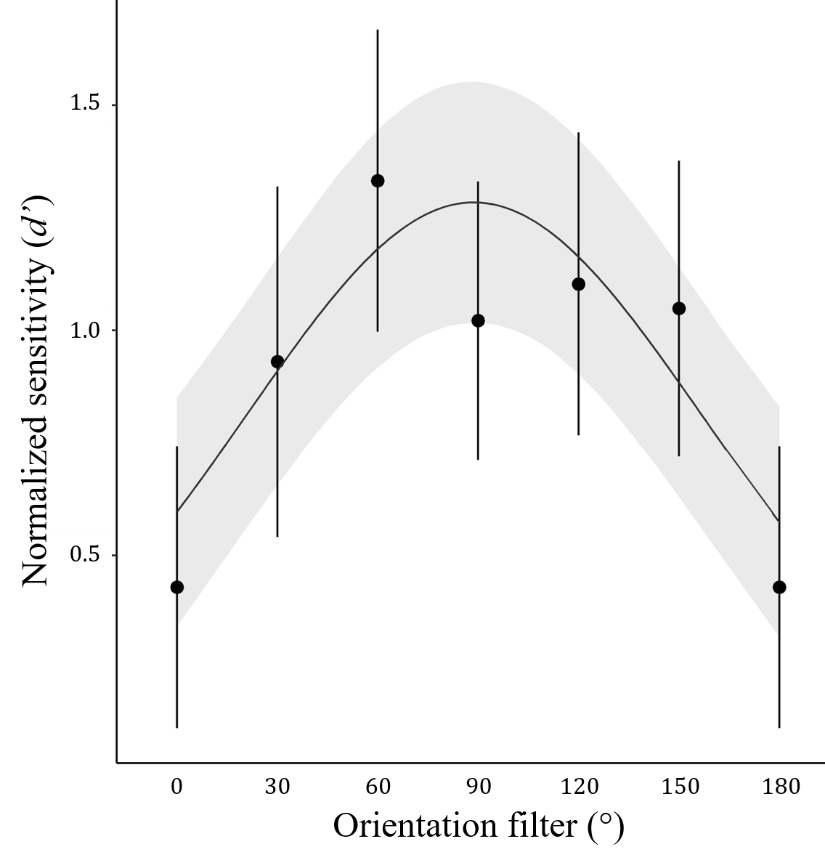
In brief, recognition performance peaked in the horizontal range (Peak location estimated at 88.8°) in line with previous studies (e.g., Goffaux & Greenwood, 2016). However, the tuning was shallower than in previous work, with a mean standard deviation estimate at 70.1°. We noticed that the highest performance was obtained in the close-to-horizontal oblique orientation filters (60° and 120°) with an advantage in the left oblique range relative to the right oblique. Previous studies, like Dakin and Watt (2009) or Goffaux and Greenwood (2016), also found a slight asymmetry of the orientation tuning in favor of one of the left oblique filtering condition . When looking at our individual data (Supplementary 2), some participants show similar asymmetry in favor of one or the other oblique condition. This asymmetry may result from an adaptation to natural face statistics. Indeed, despite the human face anatomical symmetry, its appearance in everyday life is not. Non-frontal pose, illumination direction typically from above, and tilts relative to the observer may tune the face-selective visual system to one oblique orientation more than to the other, symmetrical one. The fact that we used ‘ambient’ images of celebrities, i.e., images of faces shot under a variety of poses and illumination directions, may have
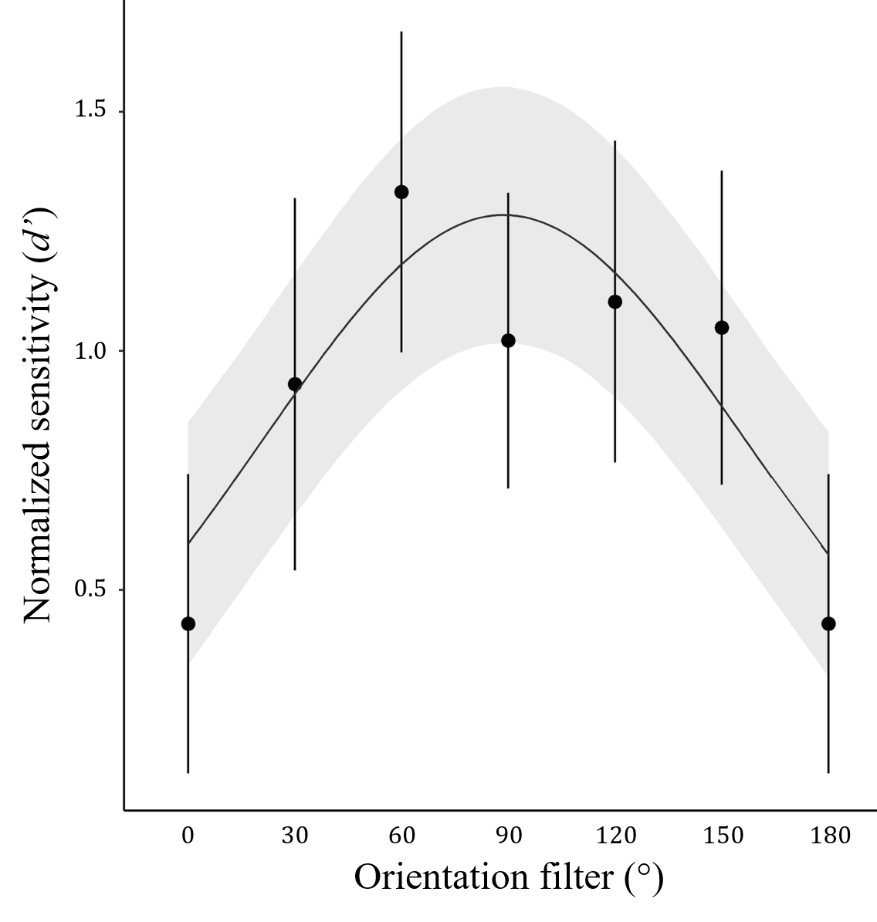
amplified this asymmetry in the recognition performance.

**Figure S.2**. Population-level averaged recognition performance in the Natural face condition and Bayesian Gaussian mixed model predictions. The dots are the population-level averaged normalized sensitivity with the 95% confidence interval in error bars. The mean of the model posterior predictions is drawn in black (line) and the 89% credible intervals of the predictions are represented as faded ribbons.

**Table S.2.** Credible interval (CrI), median and mean estimates of the four parameters of the Bayesian Gaussian model of the recognition performance in the Natural face condition.

| **Parameter** | **CrI_low** | **CrI_high** | **Median** | **Mean** |
| --- | --- | --- | --- | --- |
| Peak Location | 81.32 | 96.18 | 88.82 | 88.76 |
| Standard Deviation | 52.68 | 87.9 | 69.78 | 70.09 |
| Base Amplitude | -0.45 | 0.51 | 0.04 | 0.02 |
| Peak Amplitude | 0.8 | 1.71 | 1.24 | 1.27 |

S.5 - Comparison of the inversion and negation effects for full spectrum faces and orientation-filtered faces

Inversion and negation presumably disrupt distinct aspects of face processing, yet we found that their orientation tuning profiles were highly correlated. We investigated whether the high similarity in orientation tuning is due to these effects being highly correlated in full spectrum conditions, which would question their supposed relative functional independence (as reported in previous studies, e.g., (Bruce & Langton, 1994; Hole et al., 1999; Itier & Taylor, 2004; Kemp et al., 1990; Liu-Shuang et al., 2015)).

To this aim, we first analyzed the correlation between inversion and negation effects for full spectrum images. The correlation of the full spectrum inversion and negation *d’ effects* was moderate and non-significant using a frequentist correlation method (r = .43, p = 0.051, CI = [-.001, .727]), as well as when using a Bayesian correlation analysis (r = .33; CI = [-.03, .63]; and BF = 2.11). As a comparison, we computed the same correlation between the inversion and negation *d’ effects* averaged across orientations (r=.61, p<.001, CI = [.495, .701]). However, these results need to be interpreted cautiously as the lack of significance might be due to a lack of statistical power; indeed, compared to other correlation analyses reported in this work, the number of trials on which the present analysis relies is quite small (6 times lower in the full spectrum condition than in all filtered conditions combined).

Yet, these results suggest that while their shared orientation tuning profile inversion and negation effects seem decorrelated in full spectrum viewing conditions.

Furthermore, as we measured a stronger inversion effect compared to the negation effect for filtered images, we wanted to measure whether this difference was found for full spectrum images as well. To this aim, we first compared the recognition performance for full spectrum in inverted *vs* negated faces, using a Bayesian general linear mixed model (GLMM), using the participants as a random effect to consider the inter-subject variability in the estimations, with this equation:

sensitivity (*d’*) ~ Stimulus type + (Stimulus type | Subject)

We ran the model with default *brms* priors with 4 Markov chain Monte Carlo (MCMC) with 6,000 iterations each (2,000 warm up, resulting in 16,000 iterations after warmup), and evaluated the fit with diagnostic values such as the number of divergent chains post warmup, the Rhat statistics and the effective sample size the convergence of the chains.

We found no difference in recognition performance between inverted and negated full spectrum faces: Inversion (mean = .72 , CrI = [.37, 1.06]) , Negation (mean = .58 , CrI = [ .22, .93]) ( see Fig. S.3). In other words, we did not replicate the difference in effect magnitude between inversion and negation that we found with orientation-filtered images.


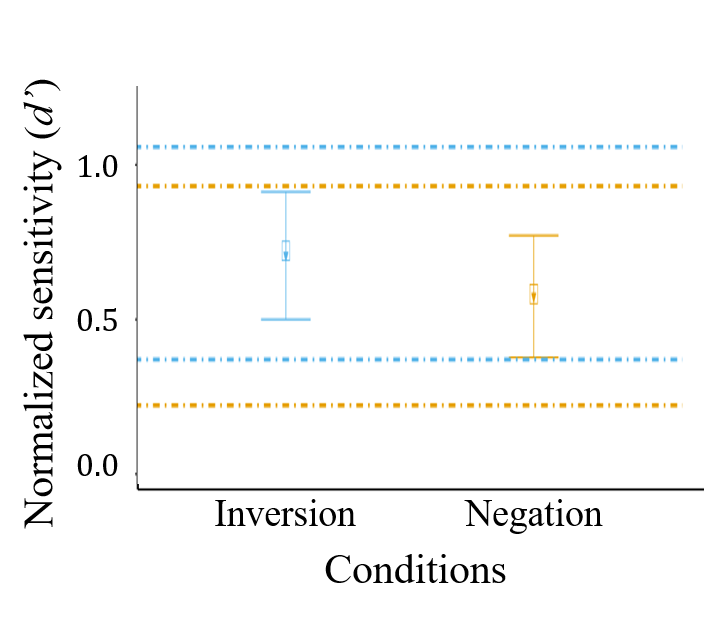


**Figure S.3**. Predictions from the GLMM of the recognition performance for full spectrum inverted and negated faces. The overlapping 89% CrI of model predictions and the similar values indicate that both Stimulus types gave similar recognition performance when the images were full spectrum. The triangles and error bars represent the mean and standard error of the performance in each condition from real data. The square is the mean in each condition given by the model prediction, and the doted lines are the 89% CrI.

S.6 – Accuracy analysis

Although sensitivity (*d’*) analysis is the most adapted to our data, we have repeated our analysis on accuracy (proportion correct) to have a better appreciation of the data. Data was processed as described in the manuscript in the Material and Methods>Data Analysis section. Mean accuracy in the Natural condition was close to ceiling (.94 ± 0.02; Figure S.4). The mean accuracy for the Inverted and Negated conditions was well above chance level (Inverted = .78 ± 0.03; Negated = .85 ± 0.03), which was also the case for every filter condition (see Figure S.4 and Table S.3 below).


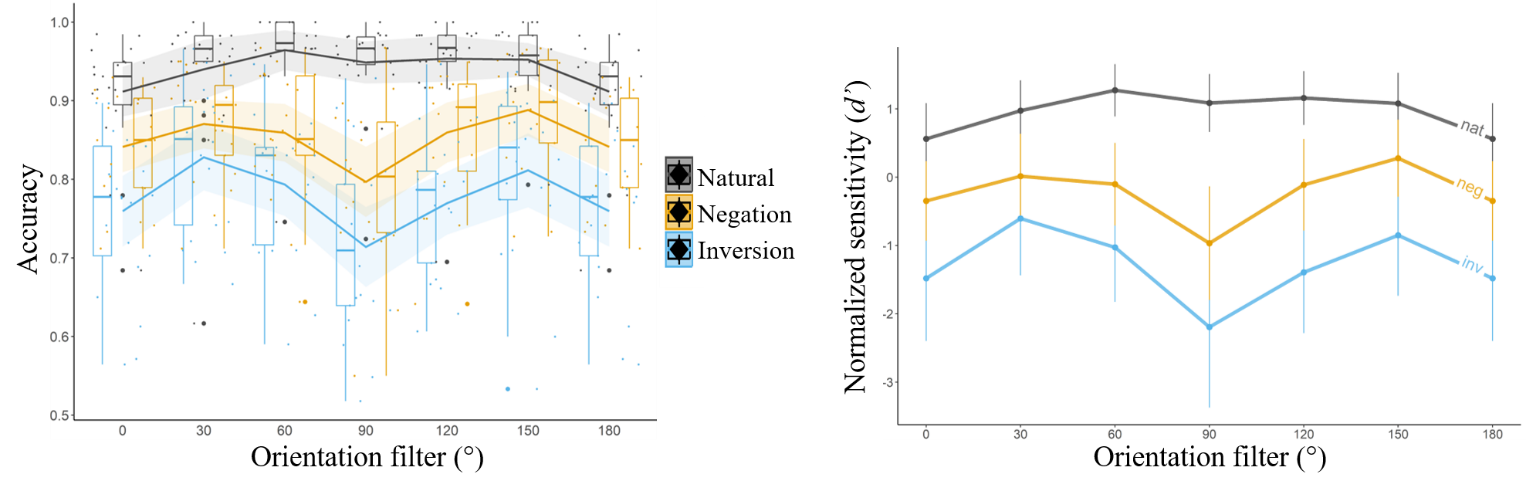


**Figure S.4.** **Left**: Averaged accuracy (proportion correct) data for each Orientation filter in each Stimulus type condition. The lines link mean accuracy values across orientation filters, boxplots represent the 25%, 50% (median) and 75% quantiles. Each point is one participant’s recognition performance for an Orientation filter and Stimulus type. **Right**: Group-level normalized accuracy for each Orientation filter in each Stimulus type condition (mean and 95% CI).

**Table S.3**. Accuracy (proportion correct) in each experimental condition averaged across participants.

| **Cond** | **All im** | **Filt. only** | **FS** | **0°** | **30°** | **60°** | **90°** | **120°** | **150°** |
| --- | --- | --- | --- | --- | --- | --- | --- | --- | --- |
| natural | .94 | .94 | .97 | .91 | .94 | .96 | .95 | .95 | .95 |
| negated | .86 | .85 | .94 | .84 | .87 | .86 | .80 | .86 | .89 |
| inverted | .80 | .78 | .94 | .76 | .83 | .79 | .71 | .77 | .81 |

There were significant differences in recognition performance between Stimulus type conditions. On average, (normalized) accuracy was better for natural than inverted and negated faces, and for negated than inverted faces (see average accuracy and normalized accuracy data in the Figure S.4; Natural CrI = [.64; 1.35]; Inverted CrI = [-1.8; -.57]; Negated CrI = [-.64; .35]). Inversion and negation effects on accuracy were bell-shaped as found with sensitivity d’, peaking around 90° (horizontal filter), and could be modelled with the same Gaussian model than the *d’* data. Parameter estimates of this model are reported in Table S4.

**Table S.4.** Summary of the population-level parameter estimates of the Gaussian mixed model run on normalized accuracy data (dependent variable is normACC effect; i.e. difference in normalized accuracy between the natural and inverted conditions and between the natural and negated conditions). The 89% credible interval (CrI) of the Base Amplitude parameter on inversion and negation effects do not overlap indicating a significant difference between the two conditions.

|  | **Inversion Effect** | | | | **Negation Effect** | | | | |
| --- | --- | --- | --- | --- | --- | --- | --- | --- | --- |
| **Parameter** | **Median** | **Mean** | **CrI lower** | **CrI upper** | **Median** | **Mean** | **CrI lower** | **CrI upper** | |
| Peak Location | 93.74 | 93.7 | 89.60 | 97.8 | 89.86 | 89.88 | 84.02 | 95.74 |  |
| Standard Deviation | 28.3 | 28.4 | 22.05 | 34.11 | 27.41 | 27.56 | 18.98 | 35.91 |  |
| Base Amplitude | 1.71 | 1.71 | 1.38 | 2.02 | .81 | .81 | .53 | 1.1 |  |
| Peak Amplitude | 1.24 | 1.24 | .81 | 1.66 | 1.09 | 1.1 | .74 | 1.45 |  |

The correlation analyses on the group (i.e. participant)-level Gaussian parameters’ posterior predictions indicated that the inversion and negation effects were functionally related at the individual participant level, although the correlations were more moderate than those obtained with *d’* (Peak location r = .85, p <.0001; Peak amplitude r = .80, p <.0001; Standard deviation r = .98, p <.0001; Base amplitude r = .57, p <.01; see also scatterplots in the Figure S.5).


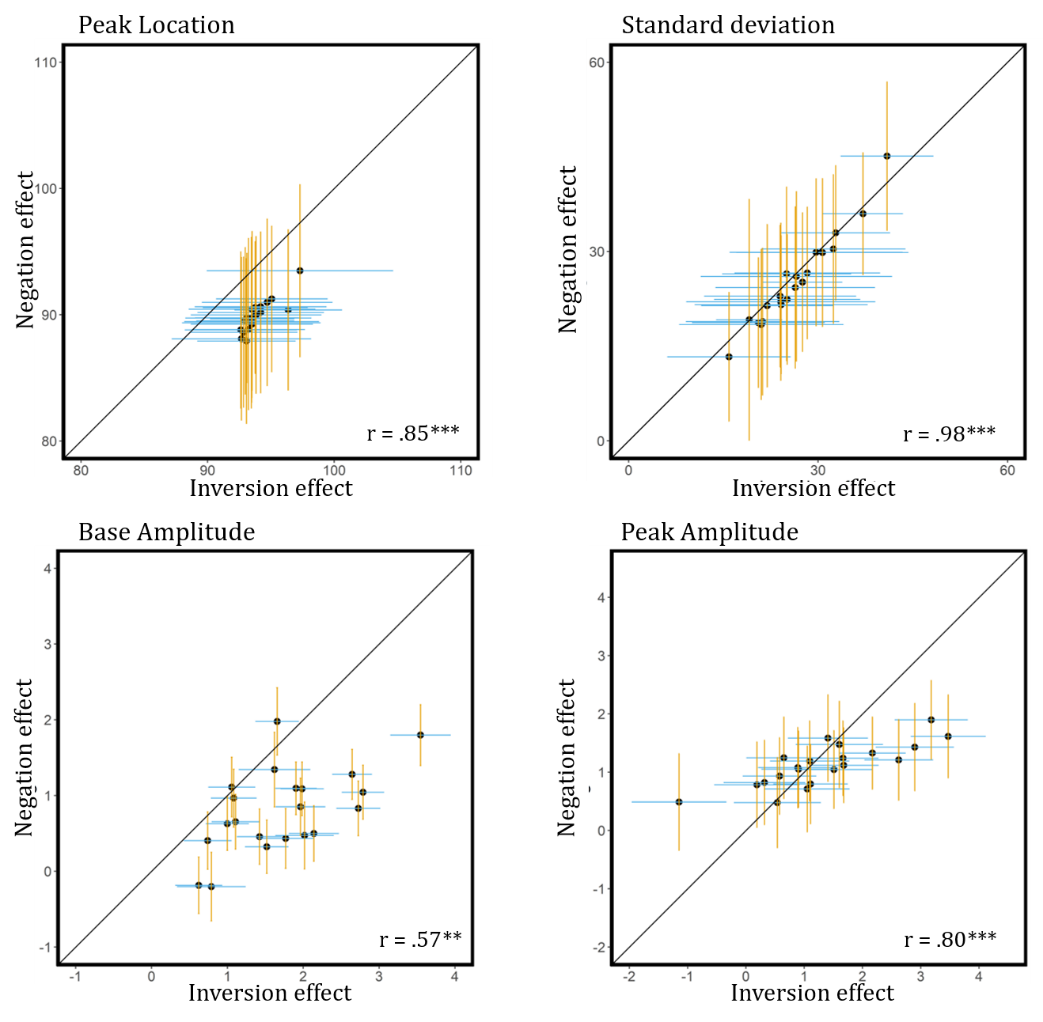


**Figure S.5**. Correlation between the group (i.e. participant)-level Gaussian parameters’ posterior predictions – from the Bayesian gaussian mixed-model run on normalized accuracy data. The error bars are the standard deviation for each group-level parameter estimate; in blue for inversion and in yellow for negation.

S.7 – Linear Mixed Modelling of Normalized sensitivity

Here we consider the possibility that the Gaussian fitting used in our main analyses may have acted as a lowpass filter of the inversion and negation effect orientation tuning profiles. Fitting the data with a Gaussian as we did may have attenuated finer differences across orientations, therefore inflating the similarity of the orientation profiles of the inversion and negation effects. We believe this unlikely accounts for the similar orientation tuning profile found for the inversion and negation effects. Indeed, the diagnostic values showed that our model made a good prediction of the data: no post warmup divergent chain, all Rhat < 1.01, all Effective sample size > 450 (see also posterior predictive check graph in Figure S6). Moreover, when fitting a simple linear mixed model to the data (without the Gaussian fit) to test the interaction between Stimulus type and Orientation filter, we find significant main effects of Stimulus type (F(1,260) = 73.67, *p* < .001) and Orientation filter (F(6,260) = 16.18, *p* < .001) but no interaction effect between the two factors (F(6,260) = 0.57, *p* = .75). These results agree with our finding from our Gaussian model that both inversion and negation effects are
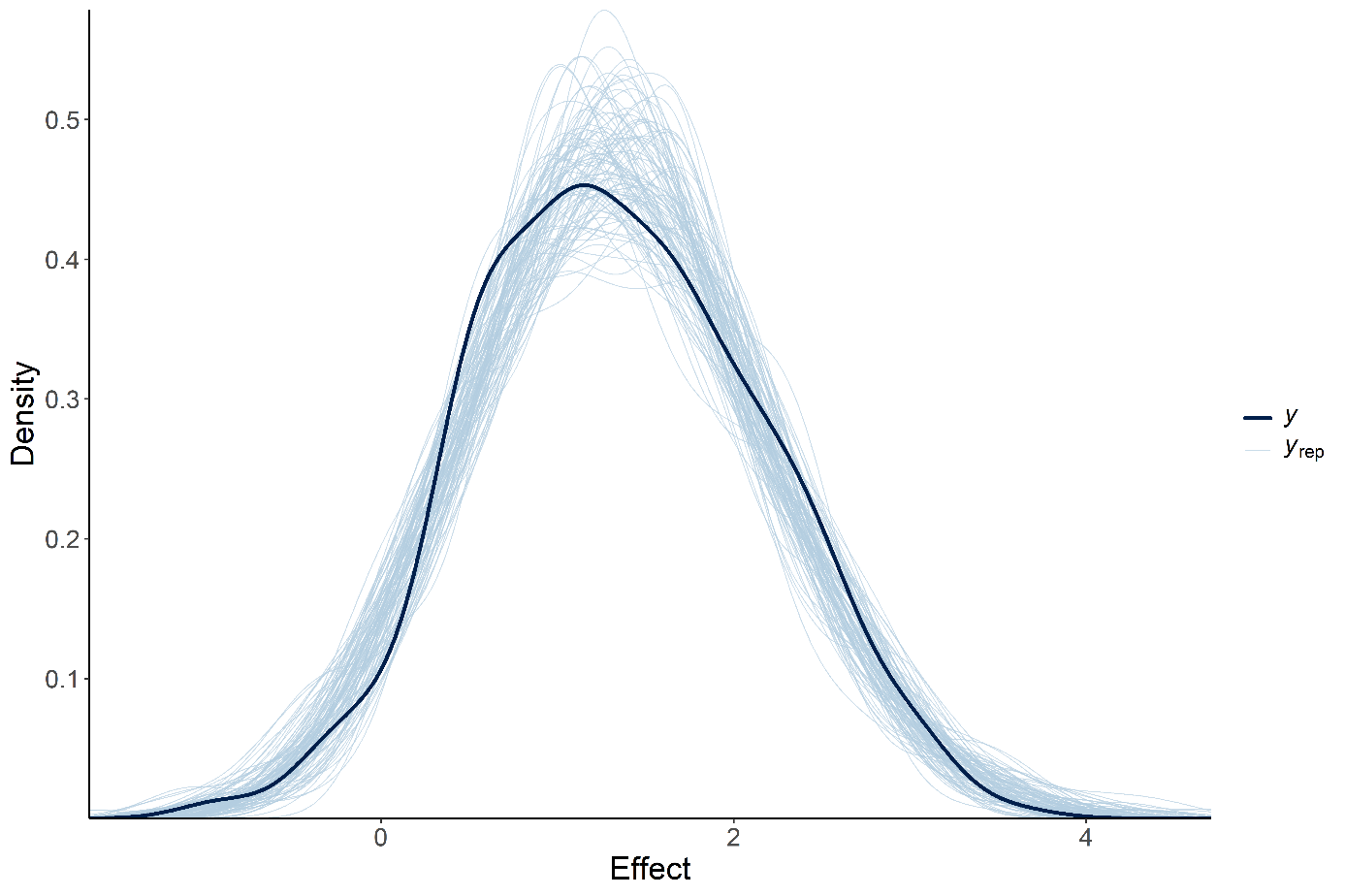
similarly disruptive across orientations.

**Figure S.6**. Posterior predictive check. The model predictions (thin light blue lines, N=100) follow the same distribution than our data (thick dark blue line), demonstrating our model has satisfactory predictive capabilities.

References

Bruce, V., & Langton, S. (1994). The Use of Pigmentation and Shading Information in Recognising the Sex and Identities of Faces. *Perception*, *23*(7), 803‑822. https://doi.org/10.1068/p230803

Dakin, S. C., & Watt, R. J. (2009). Biological « bar codes » in human faces. *Journal of Vision*, *9*(4), 2.1-10. https://doi.org/10.1167/9.4.2

DeGutis, J., Wilmer, J., Mercado, R. J., & Cohan, S. (2013). Using regression to measure holistic face processing reveals a strong link with face recognition ability. *Cognition*, *126*(1), 87‑100. https://doi.org/10.1016/j.cognition.2012.09.004

Goffaux, V., & Greenwood, J. A. (2016). The orientation selectivity of face identification. *Scientific Reports*, *6*, 34204. https://doi.org/10.1038/srep34204

Hole, G. J., George, P. A., & Dunsmore, V. (1999). Evidence for Holistic Processing of Faces Viewed as Photographic Negatives. *Perception*, *28*(3), 341‑359. https://doi.org/10.1068/p2622

Itier, R. J., & Taylor, M. J. (2004). Face Recognition Memory and Configural Processing : A Developmental ERP Study using Upright, Inverted, and Contrast-Reversed Faces. *Journal of Cognitive Neuroscience*, *16*(3), 487‑502. Journal of Cognitive Neuroscience. https://doi.org/10.1162/089892904322926818

Kemp, R., McManus, C., & Pigott, T. (1990). Sensitivity to the Displacement of Facial Features in Negative and Inverted Images. *Perception*, *19*(4), 531‑543. https://doi.org/10.1068/p190531

Liu-Shuang, J., Ales, J., Rossion, B., & Norcia, A. M. (2015). Separable effects of inversion and contrast-reversal on face detection thresholds and response functions : A sweep VEP study. *Journal of Vision*, *15*(2), 11. https://doi.org/10.1167/15.2.11
