# Supplementary material for: Horizontal face information is the main gateway to the shape and surface cues to familiar face identity
Source: PLoS One. 2024 Oct 7;19(10):e0311225. doi: 10.1371/journal.pone.0311225 (PMC11458052; doi:10.1371/journal.pone.0311225)
Supplement: Attachment — Submitted filename: Response to Reviewers.docx [file pone.0311225.s002.docx]

We thank the Editor as well as the two Reviewers for their insightful evaluation of our manuscript. We address the concerns and questions of each of the Reviewers below. Reviewers’ comments are in grey, our responses are in black, and we draw the Reviewers’ attention to important revisions made to the manuscript. As we revised extensively the manuscript, keeping all changes to the manuscript tracked automatically results in a very messy document. We therefore decided to highlight the most significant revisions by underlining them in the Revised Manuscript with Track Changes.

# Replies to reviewer #1’s comments

1. Upright accuracy is near ceiling at 93%. What are the accuracies for inverted and negated? How far from floor are they? While the choice to analyse d-prime makes sense, an additional analysis of accuracy would help readers better appreciate the data.

Mean accuracy in the Natural condition was indeed close to ceiling (.94 ± 0.02; Figure R1). The mean accuracy for the Inverted and Negated conditions was well above chance level (Inverted = .78 ± 0.03; Negated = .85 ± 0.03), which was also the case for every filter condition (see Figure R1 and Table R1 below).

**Figure R1**. **Left**: Averaged accuracy (proportion correct) data for each Orientation filter in each Stimulus type condition. The lines link mean accuracy values across orientation filters, boxplots represent the 25%, 50% (median) and 75% quantiles. Each point is one participant’s recognition performance for an Orientation filter and Stimulus type. **Right**: Group-level normalized accuracy for each Orientation filter in each Stimulus type condition (mean and 95% CI).

**Table R1**. Accuracy (proportion correct) in each experimental condition averaged across participants.

| **Cond** | **All im** | **Filt. only** | **FS** | **0°** | **30°** | **60°** | **90°** | **120°** | **150°** |
| --- | --- | --- | --- | --- | --- | --- | --- | --- | --- |
| Natural | .94 | .94 | .97 | .91 | .94 | .96 | .95 | .95 | .95 |
| Negated | .86 | .85 | .94 | .84 | .87 | .86 | .80 | .86 | .89 |
| Inverted | .80 | .78 | .94 | .76 | .83 | .79 | .71 | .77 | .81 |

We found very similar results as with *d’*, that is:

(1) On average, (normalized) accuracy was better for natural than negated faces, and for negated than inverted faces (see average accuracy and normalized accuracy data in the Figure R1 above), and there were significant differences in recognition performance between Stimulus type conditions (Inverted credible interval = [-1.8; -.57]; Negated CrI = [-.64; .35]; Natural CrI = [.64; 1.35]);

(2) Inversion and negation effects were bell-shaped, peaking around 90° (horizontal filter), and could be modelled with the same Gaussian model than the d’ data. Parameter estimates of this model are reported in Table R2.

**Table R2.** Summary of the population-level parameter estimates of the Gaussian mixed model run on normalized accuracy data (dependent variable is *normACC effect;* i.e. difference in normalized accuracy between the natural and inverted conditions and between the natural and negated conditions). The 89% credible interval (CrI) of the Base Amplitude parameter on inversion and negation effects do not overlap indicating a significant difference between the two conditions.

|  | **Inversion Effect** | | | | **Negation Effect** | | | | |
| --- | --- | --- | --- | --- | --- | --- | --- | --- | --- |
| **Parameter** | **Median** | **Mean** | **CrI lower** | **CrI upper** | **Median** | **Mean** | **CrI lower** | **CrI upper** | |
| Peak Location | 93.74 | 93.7 | 89.60 | 97.8 | 89.86 | 89.88 | 84.02 | 95.74 |  |
| Standard Deviation | 28.3 | 28.4 | 22.05 | 34.11 | 27.41 | 27.56 | 18.98 | 35.91 |  |
| Base Amplitude | 1.71 | 1.71 | 1.38 | 2.02 | .81 | .81 | .53 | 1.1 |  |
| Peak Amplitude | 1.24 | 1.24 | .81 | 1.66 | 1.09 | 1.1 | .74 | 1.45 |  |

(3) The correlation analyses on the group (i.e. participant)-level Gaussian parameters’ posterior predictions indicated that the inversion and negation effects were functionally related at the individual participant level, although the correlations were more moderate than those obtained with *d’* (Peak location r = .85, p <.0001; Peak amplitude r = .80, p <.0001; Standard deviation r = .98, p <.0001; Base amplitude r = .57, p <.01; see also scatterplots in the Figure R2 below).

**Figure R2.** Correlation between the group (i.e. participant)-level Gaussian parameters’ posterior predictions – from the Bayesian Gaussian mixed-model run on normalized accuracy data. The error bars are the standard deviation for each group-level parameter estimate; in blue for inversion and in yellow for negation.

We agree that a visibility on accuracy data is important for the reader to assess data quality, and we now report the analysis of accuracy data in the Supplementary material S6.

2. Mike Burton and others have shown that understanding how different images of the same person can vary (telling faces together) is just as important as understanding image differences between people (telling faces apart). I think the authors can contribute to this issue by analysing same vs different trials separately and testing for potential interactions with orientation filter, image manipulation, or both.

This is indeed an interesting inquiry that necessitates analysing *same* and *different* trials separately. As this analysis was not our first intent, the experiment was underpowered to test this hypothesis as indicated by the fact that the Bayesian Gaussian mixed model could not converge when considering Trial type as a fixed factor – trial number per condition being divided by 2. Therefore, we here below present the results of our exploratory separate analysis of *same* and *different* trials but prefer not to include it in the revised manuscript.

We first made sure that accuracy in *same* and *different* trials was not contaminated by biases towards one or the other response by measuring the response criterion (C) in each experimental condition using the following formula

C = - [z(H) + z(F)]/2

with H as the Hit rate and F as the False alarm rate. As all participants had |C|< .5 (i.e. unbiased responses), we pursued our analysis including all participants.

Accuracy data was mostly affected by inversion and negation in the horizontal range, whether participants had to tell two faces apart (*different* trials) or when they had to tell two faces together *(same* trials; see Figure R3 below). Interestingly, the recognition performance with vertically filtered images was more strongly disrupted by both inversion and negation in *different* than *same* trials.

**Figure R3**. Accuracy as a function of Trial type (same, different), Stimulus type (natural, inverted, negated), and Orientation filter. The lines link mean accuracy values across orientation filters, boxplots represent the 25%, 50% (median) and 75% quantiles. Each point is one participant’s recognition performance for a given experimental condition.

We tested the effect of Trial type as well as its interaction with our factors of interest with a standard linear mixed model including Trial type (same, different), Stimulus type (natural, inverted, negated), and Orientation filter as fixed effects. The model also included intercepts and slopes for the three factors and their interaction per participant as random effects (i.e. maximal random effects structure). There was a main effect of Trial type (F = 13.61, p<.001), a slight interaction with Orientation filter (F = 2.69, p<.05) and with Orientation filter and Stimulus Type (F = 1.83, p<.05). Trial type did however not interact with Stimulus type (F = 1.49, p = .23) alone.

This analysis suggests that the oriented content, and the cues disturbed by either inversion or negation, seem be differently engaged for deciding that two images of a face are from the same person than for deciding that two face images are two different individuals. This interesting question should be investigated with adequate experimental design and number of trials in future research.

3. Neither configural or surface cues were directly manipulated, and the relationships between inversion/configuration and between negation/surface are not straightforward (see Sormaz et al 2013 for example). I suggest using inversion and negation in the title and discussing the configural and surface cues in the intro and discussion.

We thank both reviewers for calling our attention on this theoretical point.

Inversion is thought to disrupt the ability to perceive the face (shape) properties as a whole (i.e., holistic processing)(Bozana Meinhardt-Injac, Malte Persike, Günter Meinhardt, 2013; Jiang et al., 2011; Rossion, 2009). The effect of negation has been attributed to the disrupted processing of surface cues (Bruce & Langton, 1994; Kemp et al., 1996; Liu et al., 2000; Russell et al., 2006; Vuong et al., 2005). The evidence as to whether such surface cues contribute to holistic mechanisms as strongly as shape cues is so far mixed (Hole et al., 1999; Sormaz et al., 2013; Taubert & Alais, 2011). Yet the disruption of holistic processing both for inverted and negated faces as shown by some recent studies (Bozana Meinhardt-Injac, Malte Persike, Günter Meinhardt, 2013; Jiang et al., 2011; Sormaz et al., 2013) indicates that these effects may modulate partially overlapping functions linked to holistic face processing. This consideration is in our view out of the scope of the present study as we base our approach on the well accepted notions that (a) inversion alters the shape properties of the face; (b) negation flips the gradient of the face surface while leaving shape cues unchanged, and (c) inversion and negation carry distinct sources of information to holistic processing. We agree with the Reviewer that the use of the term ‘configuration’ was misleading. Indeed, whether the holistic processing of face shape uniquely relies on face configuration or involves other shape cues is elusive and not the scope of this paper. We revised the title and whole manuscript extensively. We removed the term ‘configuration’ from the revised manuscript, and replaced it by the broader, and less loaded ‘shape’ term*.*

In short, the new version stays closer to what can be asserted with greater confidence regarding inversion and negation: inversion turns edge-related cues upside-down whereas negation flips the luminance gradient of the face surface and preserves face edges.

4. Supplementary analysis 5 is useful but the current interpretation is based on an indirect contrast between a significant correlation (.61) and a non-significant one (.41). These correlations might not differ statistically, so the authors need to compare them directly (see [http://quantpsy.org/corrtest/corrtest2.htm](https://eur03.safelinks.protection.outlook.com/?url=http%3A%2F%2Fquantpsy.org%2Fcorrtest%2Fcorrtest2.htm&data=05%7C02%7Chelene.dumont%40uclouvain.be%7C338d141529eb43198bdc08dc94475797%7C7ab090d4fa2e4ecfbc7c4127b4d582ec%7C1%7C0%7C638548282143242733%7CUnknown%7CTWFpbGZsb3d8eyJWIjoiMC4wLjAwMDAiLCJQIjoiV2luMzIiLCJBTiI6Ik1haWwiLCJXVCI6Mn0%3D%7C0%7C%7C%7C&sdata=Ow5sll16l34oU%2FUI65InJd2qZsCwhMDH7H19tT54RPs%3D&reserved=0)) and adjust their interpretations if needed.

The objective of this supplementary analysis was not to compare effect correlations across full spectrum and orientation-filtered conditions. Our goal was to verify the independence of inversion and negation effects for full spectrum images in line with past evidence (e.g., additivity between the two behavioural effects reported by Bruce & Langton, 1994, functional selectivity of the neural effects reported by Liu-Shang et al., 2015; see more references below).

Our results tend to support the independence of inversion and negation effects in the full spectrum condition. Indeed, we did not find any evidence for a correlation between inverted and negated performance in the full-spectrum, non-filtered condition, neither with a frequentist correlation analysis (r = .43, p = 0.051) nor with a Bayesian correlation analysis (r = .33; CI = [-.03, .63]; and BF = 2.11). We however note that the lack of significance might also be due to the number of trials which is quite small (6 times lower in this full spectrum condition than in all filtered conditions combined).

Because we think that the comparison of correlations suggested by the Reviewer would be informative, we now report the confidence intervals of both correlations in S5 (full spectrum images: r = .431, CI = [-.001, .727]; filtered images: r = .609, CI = [.495, .701]). While the confidence interval for the filtered images correlation was well above 0, it overlapped with the interval of the full spectrum correlation. We therefore cannot conclude that the correlation is significantly stronger in filtered than full spectrum conditions.

The Steiger test suggested by the reviewer is not adequate because it is used to compare correlations sharing a common variable, which was not the case here (the two correlations are independent).

We have clarified the objective of this analysis and added the Bayesian correlation analysis and the confidence intervals around the correlation for full spectrum and filtered conditions in the revised Supplementary S5. We now explicitly acknowledge that our evidence for the inversion/negation effects independence is rather limited.

# Replies to reviewer #2’s comments

I have a mostly favourable opinion of this work, which has generally sound methodology and statistical analyses.

We thank the reviewer for their positive feedback.

However, I do take issue with some interpretations and general framing of the paper (immediately below). I also have a few requests for clarification and recommendations.

The authors begin with a claim that the cause for human reliance on horizontal facial information is not well understood and that the visual system adapting to configuration and surface cues might explain this (e.g., lines 24-25). This framing hinges on 1) face inversion and contrast reversal effects being valid measures of each construct, and 2) on each construct being empirically distinct. However, disruption of configural processing is only one possible explanation—though one often shared—for the face inversion effect, for instance. But there are alternatives.

We thank both reviewers for calling our attention on this. We have changed our title (now “Horizontal face information is the main gateway to the shape and surface cues to familiar face identity”) and revised the Introduction and Discussion sections to account for this comment.

Inversion is thought to disrupt the ability to perceive the face (shape) properties as a whole (i.e., holistic processing)(Bozana Meinhardt-Injac, Malte Persike, Günter Meinhardt, 2013; Jiang et al., 2011; Rossion, 2009). The effect of negation has been attributed to the disrupted processing of surface cues (Bruce & Langton, 1994; Kemp et al., 1996; Liu et al., 2000; Russell et al., 2006; Vuong et al., 2005). The evidence as to whether such surface cues contribute to holistic mechanisms as strongly as shape cues is so far mixed (Hole et al., 1999; Sormaz et al., 2013; Taubert & Alais, 2011). Yet the disruption of holistic processing both for inverted and negated faces as shown by some recent studies (Bozana Meinhardt-Injac, Malte Persike, Günter Meinhardt, 2013; Jiang et al., 2011) indicates that these effects may modulate partially overlapping functions linked to holistic face processing. This consideration is in our view out of the scope of the present study as we base our approach on the well accepted notions that (a) inversion alters the shape properties of the face; (b) negation flips the gradient of the face surface while leaving shape cues unchanged, and (c) inversion and negation carry distinct sources of information to holistic processing. We agree with the Reviewer that the use of the term ‘configuration’ was misleading. Indeed, whether the holistic processing of face shape uniquely relies on face configuration or involves other shape cues is elusive and not the scope of this paper. We revised the title and whole manuscript extensively. We removed the term ‘configuration’ from the revised manuscript, and replaced it by the broader, and less loaded “shape” term*.*

In short, we agree that we have placed too much reliance on inversion and negation as valid constructs of configuration and shading cues. The new version stays closer to what can be asserted with greater confidence about inversion and negation: inversion turns edge-related cues upside-down whereas negation flips the luminance gradient of the face surface and preserves face edges.

Furthermore, the authors support their independence hypothesis with the results of a correlation between face inversion and contrast reversal effects for full spectrum faces being nonsignificant. While I appreciate that, strictly speaking, a correlation of r = 0.43, p = 0.051, is not statistically significant, this is not exactly evidence of their independence.

We agree that the absence of correlation cannot be taken as strong evidence that the inversion and negation effects are decorrelated in full spectrum viewing conditions.

Since (1) the correlation is non-significant in the full spectrum condition, (2) the confidence interval of this correlation include zero but only by a narrow margin (r = .431; CI = [-.001, .727]), and (3) a Bayesian correlation analysis does not allow to conclude on either a correlation nor for an absence of correlation (r = .33; CI = [-.03;.63]; Bayes Factor = 2.11), we now explicitly acknowledge that our evidence for the inversion/negation effects independence is rather limited. Note that this correlation of the two effects in the full-spectrum condition relies on a moderate number of trials (60 for each Stimulus Type), which may have caused a lack of statistical power.

Following Reviewer 1’s suggestion, we revised S5 and now provide the confidence intervals of the inversion/negation effect correlation in full spectrum and filtered conditions as well as their Bayes Factors. We also acknowledge that the correlation analysis in full spectrum condition is likely underpowered.

Furthermore, the fact that orientation profile modeling parameters of the face inversion and contrast reversal effects correlated almost perfectly suggests these processes do share some fundamental aspects—though admittedly this correspondence was likely inflated vs. the true correlation, due to the smooth nature of Gaussian fitting (see also below). Nevertheless, my criticism essentially boils down to this: Configuration and surface cues are theoretically loaded constructs that are assumed to be altered in the face inversion and contrast reversal effects respectively. Prefacing horizontal tuning with these does nothing to enrich the conversation—and potentially even derails it in light of the points I raised above. An alternative and perhaps better framing is that researchers have long been trying to explain face inversion and contrast reversal effects. Though a disruption of configuration and surface cue processing are possible explanations, there are alternatives. With the recent surge in studies suggesting horizontal tuning is a core feature of the face processing architecture, the authors verified whether and how face inversion and contrast reversal disrupt orientation tuning. This more neutral framing benefits from its lack of assumptions regarding face inversion and contrast reversal effects and adds to a growing literature that shows the functional importance of horizontally orientated information for various aspects of face processing.

We thank the Reviewer for their suggestion. This is indeed another possible framing of our research question. However, there is numerous evidence that the horizontal face information is crucial to face identity recognition, but the nature of the information carried by this range is unknown. We thus believe that using inversion and negation to address this question is important to move forward our understanding of the visual information driving the recognition of human face identity.

Therefore, we think it is worth prefacing the Introduction of this manuscript with the horizontal tuning as a core property of face perception, as this was our entry point to this study. The study aimed to better understand the horizontal tuning of face recognition by looking at how the two commonly studied image manipulations known to disrupt distinct sources of information (shape and surface, respectively), inversion and negation, alter the tuning to horizontal cues.

However, we agree that the original version of our manuscript was too confident about the theoretical implications of inversion and negation effects. As mentioned in our response above, we thus revised our manuscript following the suggestion of this Reviewer and another Reviewer to reconsider these strong theoretical assumptions, in particular the link we made in the original manuscript between inversion and ‘configuration’. We hope that with the revision, the rationale behind the study is now clearer and more neutral (less engaged theoretically).

Writing is generally good, but the authors should watch out for overreliance on the word “the,” which at times makes reading arduous. E.g., in the sentence starting at line 289, there are 8 “the” in a single sentence, several of which are superfluous. “The purpose of this study was to understand the extent to which the horizontal tuning of face identification reflects the contribution of feature configuration or of the surface and shading of the face image through the comparison of inversion and negation effects on the Orientation filter dependence of identity recognition performance.”

We thank the Reviewer for this comment on our writing. The new version of the manuscript has been revised to improve the problem raised by the Reviewer.

Line 182: Were mean luminance and RMS contrast set before or after applying the elliptical aperture? Please make this more explicit in text.

The RMS contrast was equalized before adding the elliptical aperture, and the aperture was the same luminance as the background of the experiment (see page 7, line 182).

Line 216: Were actor identities selected on a participant basis? Please make this more explicit in text.

Before the experiment, each participant underwent a pre-screening phase in order to select the 10 actors they were most familiar with among our image set of 18 actors. Each participant then went through the main experiment with those 10 individually pre-selected actors. This aspect of the procedure is described on page 8, line 206-218.

The set of 18 actors was the same for every participant and was constituted during an online pilot phase of our experiment (on a different subject sample), as described in the Procedure section of the Methods (page 6, line 154-162) as well as in S1.

Line 261: It might benefit the reader to remind what chance level was here.

This is now clarified (page 9, line 259).

Line 305: The authors should explain why they chose to analyze normalized d’, rather than simply d’. Also, they should provide a bit more detail about how this was done, as I found myself a bit confused by their description of the procedure.

Normalized *d’* was calculated using the group-level mean and standard deviation of sensitivity *d’*, all experimental conditions combined. We used normalization to rescale our data. Such normalization procedure on dependent variables is a common practice when using complex hierarchical models, as it facilitates model convergence (Stan Development Team, 2023). This is now clarified (page 11, line 305-3012).

Line 333: Base amplitude and peak amplitude should be clearly explained here (perhaps put in relation with the data at hand). Peak amplitude is also poorly explained at line 440.

The meaning of the four parameters of the Gaussian tuning function have been clarified (page 13, line 360-364).

Line 469: The authors should consider the possibility that these coefficients are somewhat inflated due to the (smooth) Gaussian fit. They could perhaps exploratorily look at the relationship between inverted and reversed d’ as a function of orientation (perhaps as a random effect).

We agree with and thank the Reviewer for raising the possibility that the Gaussian fitting may act as a lowpass filter that would eliminate the fine differences across orientations, therefore inflating the similarity of the orientation profiles of the inversion and negation effects. After reflection, we do not think this has influenced our results first because the diagnostic of the fit shows that our model is able to make good prediction of the data (see posterior predictive check graph in Figure R4 below). Next, and in order to verify further that inversion and negation similarly recruit the oriented information, we fitted a simple linear mixed model (without the Gaussian fit) to test the interaction between Stimulus type and Orientation filter. Our results show significant main effects of Stimulus type (F(1,260) = 73.67, *p* < .001) and Orientation filter (F(6,260) = 16.18, *p* < .001) but no interaction effect between the two factors (F(6,260) = 0.57, *p* = .75). These results agree with our finding from our Gaussian model that both inversion and negation effects are similarly disruptive across orientations.

We added this information to the Results section (page 17, line 475-481), and added the analysis to Supplemental S7.

**Figure R4**. Posterior predictive check. The model predictions (thin light blue lines, N=100) follow the same distribution as our data (thick dark blue line), demonstrating good prediction from our model has satisfactory predictive capabilities.

They may also want to correlate individual full spectrum d’s with orientation d’s to look at whether one orientation is more likely to account for individual differences in performance vs. others. I am fully aware the study may be underpowered if the correlations are only small/moderate, but it might nevertheless be informative about some trend within the data (perhaps mixed effects modeling would again offset some of that).

The correlation between the performance with full spectrum images and each orientation-filtered images at each condition can be found in Table R3 below. Again, these results need to be interpreted cautiously as they are most likely underpowered.

What we mainly observe from these is that the correlation of natural images recognition performance between full spectrum and orientation-filtered conditions tends to culminate in the horizontal while performance correlations between full spectrum and inverted and negated orientation-filtered conditions do not. Furthermore, the correlation lack significance for vertical and close to vertical orientation filter with natural images. This suggests that natural face recognition is mainly driven by the horizontal range of face information.

Since these analyses are exploratory and likely underpowered, we did not include these results in the manuscript at this stage.

**Table R3.** Correlation coefficients between the performance in the full-spectrum condition and that in each orientation-filtered condition (0, 30, 60, 90, 120 and 150°) for Natural (nat), Inverted (inv), and Negated (neg) images – along with the associated confidence intervals (CI) and p-values.

| **FS** | **Cor** | **CI_Low** | **CI_High** | **p.value** |
| --- | --- | --- | --- | --- |
| nat0 | .43 | -.003 | .73 | .05 |
| nat30 | .72 | .42 | .88 | >.001 |
| nat60 | .62 | .26 | .83 | >.01 |
| nat90 | .68 | .34 | .86 | >.001 |
| nat120 | .62 | .26 | .83 | >.01 |
| nat150 | .43 | .001 | .73 | .05 |
| inv0 | .68 | .35 | .86 | >.001 |
| inv30 | .63 | .27 | .83 | >.01 |
| inv60 | .63 | .27 | .83 | >.01 |
| inv90 | .35 | -.09 | .68 | .12 |
| inv120 | .58 | .20 | .81 | >.01 |
| inv150 | .73 | .43 | .88 | >.001 |
| neg0 | .60 | .23 | .82 | >.01 |
| neg30 | .59 | .22 | .82 | >.01 |
| neg60 | .76 | .50 | .90 | >.001 |
| neg90 | .64 | .29 | .84 | >.01 |
| neg120 | .71 | .40 | .87 | >.001 |
| neg150 | .55 | .16 | .79 | >.01 |

Figure 3: plotting face inversion effect and contrast reversal effect as “normalized sensitivity (d’)” is a bit counterintuitive as it might initially suggest that sensitivity is highest with horizontal information. This could be improved by e.g., also specifying on the axis that a positive d’ value = sensitivity loss, or by plotting orientation profiles upside-down (i.e., with negative d’s).

The axis has been changed from “normalized sensitivity” to “effect”. We have also added in the caption a description of how the normalization and the effect are obtained.

Figures 3 and 4: should their order be inverted? It seems raw results (Fig 4) should be presented before fitted curves (Fig 3).

We thank the Reviewer for the suggestion, we have implemented the change in our manuscript.

Full spectrum trials might provide valuable context, e.g., by being plotted alongside orientation d’, or even plotting orientation d’ as a proportion of the full spectrum (or again, to investigate individual differences across orientations, see above point).
I think upright fitted orientation profiles belong in the main text rather than supplementary materials.

The full spectrum *d’* for each condition were added on Figure 3 (formerly referred to as Figure 4) as full lines in the background. We thank the Reviewer for this suggestion as it indeed provides valuable information.

The upright fitted orientation profile was also added to this figure (3c).

**Figure R5.a**. Group-level normalized sensitivity (*normd’*) for identity recognition as a function of Orientation filters. The black diamonds represent the mean performance for each condition and the boxplots represent the 25%, 50% (median) and 75% quantiles. Each point is one participant’s recognition performance for an Orientation filter and Stimulus type. The half violins represent the distribution of the data for each condition. The full straight lines represent the performance in full spectrum for each Stimulus Type **b**. Predictions from the Bayesian general linear mixed model comparison of Stimulus type. The non-overlapping 89% credible intervals (CrI) of model predictions indicate a substantive performance difference between each stimulus type. The triangles and error bars represent the group mean and standard error of the data in each condition. The square is the mean in each condition given by the model prediction, and the doted lines are the 89% CrI. **c**. Population-level averaged recognition performance for natural images and Bayesian non-linear mixed model predictions. The dots are the population-level averaged normalized sensitivity with the 95% CrI in error bars. The model mean posterior prediction is drawn in black (line) and the 89% CrI of the predictions are represented as faded ribbons.

Credible interval: value used (89%) should be in text (Data and analyses).

This precision has been added in the manuscript.

Regarding the broad orientation tuning found (especially for upright faces), the authors report having used a 25deg wide orientation filter. According to my calculations, adjacent orientation samples (e.g., 90deg and 120deg) were not independent with this width parameter, as they intersect at around mu +/- 15deg. Briefly, a horizontal (90deg) filter and oblique-horizontal (120deg) filter both sample 105deg information at approx. 37% of the maximum sampling proportion. The 120deg filter also samples 90deg information at 2% of the maximum sampling proportion (and inversely, the 90deg filter samples 120deg information at 2%). In fact, their respective areas overlap by about 8%. This is not a problem in and of itself, but it should be recognized by the authors, and it might partially account for somewhat wider orientation tuning found.

There is indeed an overlap in the orientations presented during our experiment as in many past studies on the orientation tuning of face perception (Dakin & Watt, 2009; Pachai et al., 2017). Indeed, the filter bandwidth needs to be sufficiently large to provide enough information to recognize face identity. In their experiment Dakin and Watt (2009) used a bandwidth of 23° and therefore presented images with a comparable overlapping oriented content to ours. Yet, the recognition accuracy they measure almost doubles between the vertical and the horizontal range. Furthermore, Pachaï et al. (2017) compared the effect of filter bandwidth on the orientation selectivity of face recognition. Using familiar faces, they measured a significant performance advantage for horizontal compared to vertical images for orientation filters with bandwidth from 15° to 90°.

In the revised Methods, we justify the bandwidth of the orientation filters and acknowledge there is a portion of shared energy across orientation filters (page 7 line 170-176). In the revised Discussion, we state that the filter bandwidth could somewhat account for the broad orientation tuning found in this study (page 19 line 542-546). We thank the Reviewer for suggesting this addition.

Supplementary materials, line 77-78 : “A hypothesis about the origin of the asymmetry is that it would result from faces statistics that often appear tilted and not perfectly upright.” Looking at Figure 1, it seems 60deg and 120deg locally enhance eye, eyebrow and mouth contrast more vs. the 90deg filter, at least subjectively. Could this be a factor, seeing as those are very important features for face processing? Also, is it possible this was caused by how luminance and RMS contrast were equated?

We agree that the eye region is peculiarly salient in the oblique ranges adjacent to the horizontal range. However, as discussed in the Supplementary materials (S4), performance was higher in the 60° filter condition than in the 120° filter condition. This asymmetry is probably not random, since we noticed that it was also observed in previous studies (Dakin & Watt, 2009; Goffaux & Greenwood, 2016). We raised the possibility that this could be due to the natural statistics of everyday face appearance. Despite the human face anatomical symmetry, its appearance in everyday life is not. Non-frontal pose, illumination direction, and tilts relative to the observer may tune the face-selective visual system to one oblique orientation more than to the other, symmetrical one. The fact that we used ‘ambient’ images of celebrities, i.e., images of faces shot under a variety of poses and illumination directions, may have amplified this asymmetry in the recognition performance. We developed the discussion of the S4 findings considering these potential accounts. The RMS contrast equalization could indeed accentuate any effect of uneven face natural statistics and exacerbate the effect. However, while a full spectrum face image contains most of its energy in the horizontal range, energy is still relatively strong in the adjacent oblique ranges. Therefore, we think that the impact of RMS equalization is relatively moderate in these ranges.
